# Effects of different thermal processing methods on the structure and allergenicity of peanut allergen Ara h 1

**DOI:** 10.1002/fsn3.742

**Published:** 2018-07-27

**Authors:** Yang Tian, Huan Rao, Ke Zhang, Sha Tao, Wen‐Tong Xue

**Affiliations:** ^1^ Beijing Advanced Innovation Centre for Food Nutrition and Human Health College of Food Science and Nutritional Engineering China Agricultural University Beijing China; ^2^ College of Food Science and Nutritional Engineering China Agriculture University Beijing China; ^3^ College of Information and Electrical Engineering Beijing China

**Keywords:** Ara h 1, peanut allergen, thermal processing

## Abstract

Boiling and frying can alter the structure of peanut allergens and therefore change the IgE‐binding capacity of the Ara h 1. In this research, we aim to clarify the connections between structural changes and the allergenicity alteration, and recommend an effective thermal method to minimize the allergenicity of Ara h 1. Anion exchange chromatography was used to isolate Ara h 1 from non/heat‐treated peanuts. Ara h 1 in boiled peanuts has a relatively low hydrophobic index, reduced maximum emission wavelength in the fluorescence, less content of α‐helix, and the lowest IgE‐binding efficiency. On the contrary, Ara h 1 in fried peanuts present a much higher degeneration degree, a red shift in fluorescence, and a decrease in the content of α‐helix. These data indicate that boiling can reduce the allergenicity of Ara h 1, thus can be utilized in peanut processing from a security point of view.

## 1 INTRODUCTION

As one of the greatest food hypersensitivity, peanut allergy has raised people's attention among the world. The prevalence of peanut allergy rises over the past 10 years (Chen, Welch, & Laubach, 2018; Shreffler et al., 2018). According to reports, 0.5% of English people were allergic to peanut, more than 0.6% of infants and 1.1% of adults were sensitive to peanut in the United States (Nwaru et al., 2014). Approximately 0.3%–4.1% of children (aged 2–5 years old) were sensitive to peanut in European countries (Shreffler et al., 2018). The classic symptoms of peanut allergy include urticarial, wheals, diarrhea, and bronchospasm (Du Toit et al., 2015; Jones et al., 2017). Most patients cannot develop resistivity to peanut. Hypoallergenic derivatives which converted from allergens are utilized as vaccines to reduce anaphylaxis (Katherine Anagnostou, 2014; Wood et al., 2013), but the best protection for allergic patients still is avoiding any peanut intake (Burks et al., 2015; Hurlburt, McBride, Nesbit, Ruan, & Maleki, 2014). However, peanut‐free diet has become a challenge because peanuts are always involved in the production process of different kinds of food. For example, crushed peanut are often used as additives to enhance the taste and smell of chocolates and cakes (Rao et al., 2016).

As the most abundant allergen existed in the peanut kernel, Ara h 1 plays an important role in the sensitized procedure. Normally Ara h 1 is presented as a trimer, every monomer has the same molecule weight, which is 63.5 kDa, is easy to degrade but has a strong allergenicity (Alves et al., 2015; Yusnawan, Marquis, & Lee, 2012). It has been reported that Ara h 1 can be recognized by 90% of patients' serum (Chruszcz et al., 2011). These data indicate that Ara h 1 is one of the main allergens existed inside of the peanut kernel.

The prevalence of peanut allergy is relatively low in China (Lee, Thalayasingam, & Lee, 2013), the reason may lays on the commonly and commercially used heat treatments, like roasting, boiling, and frying (Sayers et al., 2016). It is reported that thermal processing can influence the allergenicity of peanut allergen (Barba, Terefe, Buckow, Knorr, & Orlien, 2015; Blanc et al., 2011; Comstock, Maleki, & Teuber, 2016; Huang, Hsu, Yang, & Wang, 2014; Vissers et al., 2011). According to previous researches, Millard reaction occurred during roasting can force the allergens to become Advanced Glycosylation End Products (AGE), this modification may induce new conformational epitopes appeared on the surface of allergens and therefore increase the allergenicity of peanut (Mueller et al., 2013; Wood et al., 2013). It has been shown that Ara h 1 extracted from roasted peanut maintained higher RBL‐2H3 cell elicitation capacity than Ara h 1 extracted from boiled peanuts (Dileepan et al., 2014). The allergenicity of boiled peanut are reported to be decreased due to the diffusion loss of allergen (Comstock et al., 2016; Jiménez‐Saiz, Benedé, Molina, & López‐Expósito, 2014). Upon boiling, Ara h 1 undergoes aggregation and form branched rod‐shaped aggregates with loss of some secondary structure which caused a decrease IgE‐binding capacity of the allergen. (Blanc et al., 2011). Besides, structural modifications may also protect the allergen from digesting. By cross‐linking with glucose, allergens would possess a complex structure, which are more resistant to pepsin and trypsin, thus the epitopes have more possibility to entry the blood and cause allergy (Teodorowicz, Fiedorowicz, Kostyra, Wichers, & Kostyra, 2013).

Although there are many experiments conducted to explore the effect of heat treatment on either total allergic food or purified allergens, their values do not represent each other because of the existence of other food matrix like fat and carbohydrates. Both kinds of researches are essential for the exploration of the mechanism of desensitization.

How the thermal process regulates the immunoreactivity of Ara h 1 remained unclear. The sensitivity change of allergens are usually related to the structural modifications appeared upon heat processing. In this research, we aim to clarify the connections between heat treatment and allergenicity of Ara h 1. Anion exchange chromatography was used to purify Ara h 1 from crude, boiled, and fried peanuts. Then the structural changes and sensitivity alteration were monitored to reveal the relations between those two.

## 2 MATERIALS AND METHODS

### 2.1 Sample preparation

Peanut kernels were (a) boiled at 100°C for 20 min; (b) fried in vegetable oil at 120°C for 20 min. For each cooking method, three packages (each containing 300 g of peanut kernels) were prepared. Three kinds of peanuts (crude, boiled, and fried) were grounded with liquid nitrogen, then acetone and ethyl ether were used to remove fat alternately for four times, each time was lasted for at least 2 h. For fried peanuts, extra 4 h of degreasing were needed because there was relatively more oil stored in the peanut kernels. Defatted peanut paste was dried by airing and became peanut powder. Defatted peanut powder was then dissolved in the extract buffer (50 mM phosphate, 0.15 M sodium chloride, pH 7.2). After shook for 24 h at 4°C, the total meal was centrifuged at 4000 g for 20 min, and the supernatant was isolated and freeze‐dried at −80°C. The peanut protein powder was stored at −80°C until use.

### 2.2 Human sera

The study involved with seven patients who had been previously confirmed to be allergic to peanut and were chosen from Fourth Hospital of Hebei Medical University, Shijiazhuang, Hebei, PR China. All of the patient sera were individually tested. The sera data were presented based on the concentration of specific immunoglobulin E (IgE) to peanut allergens (Table 1). The control serum was collected from nonatopic laboratory volunteers. The negative control serum was made by seven nonatopic volunteers' serum pool. Informed consent was obtained from each volunteer, and sera from these patients were stored at −80°C until use.

**Table 1:** Description of peanut‐allergic patients

Subjects	Code	Sex/age	Allergic history	Symptoms	Peanut allergen‐specific IgE (IU/ml)
W.Q.	P1	M/26	PA, WA	Diarrhea, Urticarial, Asthma	3.8
W.Y.Z	P2	M/32	PA, LA, WA	Vomiting, Diarrhea	2.0
Z.G.	P3	F/40	PA, WA	Diarrhea	1.4
C.T.Y	P4	M/28	PA	Asthma	3.5
C.M.	P5	M/24	PA, MAA	Vomiting, Asthma	2.2
B.Q.	P6	F/30	PA, FA	Urticarial, Asthma	1.9
H.Y.	P7	M/20	PA, MAA, MIA	Diarrhea	1.4

Note. FA: fish allergy; LA: legume allergy; MAA: mango allergy; MIA: milk allergy; PA: peanut allergy; WA: wheat allergy.

### 2.3 Ara h 1 purification by anion exchange chromatography

Methods reported by Wu et al. (2014) were modified as follows: Protein powder were dissolved in double distilled water, the concentration ranges from 50 to 70 mg/ml, then the solution was filtered through 0.45 μm membrane (Millipore, Bedford, MA). A 55 ml DEAE Fast Flow column (GE Healthcare Company) was utilized to purify Ara h 1. The column was equilibrated until the baseline become smooth. Then protein sample (5% of the column volume) was loaded to the column. By continuously increasing the concentration of sodium chloride (which dissolved in the PBS as elution buffer) to 0.4 mol/L at a flow rate of 1.0 mL/min, the eluted proteins were monitored at 280 nm and then collected in the receptor machine. SDS‐PAGE and Western Blot were subsequently performed to identify the components.

### 2.4 Sodium dodecyl sulphonate polyacrylamide gel electrophoresis (SDS‐PAGE)

All protein samples were identified by reducing SDS‐PAGE. According to methods reported by Vissers et al. (2011) and Beyer et al. (2001). Ten micrograms of protein were loaded into each well, and 12% acrylamide and 4% acrylamide were used as the separating gel and stacking gel separately. The gels were dyed by Coomassie brilliant blue R250 for 40 min and bleached in destainer (7.5% HAc (v/v), 5% methanol (v/v) in water) for 12 h. BIO‐RAD GelDoc 2000 gel imaging system was used to photograph and analysis the pictures of electrophoresis results.

### 2.5 Analyses of IgE binding efficiency by Western blots

To identify the IgE‐binding ability of the purified allergens, Western blots were performed. Methods from Rao et al. (2016) and Mondoulet et al. (2005) were modified as follows. Peanut protein extracted from crude, fried, and boiled peanuts were analyzed by reducing SDS‐PAGE and then transferred to nitrocellulose membranes for 90 min at 80 V, then the membrane was placed in blocking buffer (5% (w/v) defatted milk powder dissolved in 100 mM phosphate buffer, pH 7.2) for 90 min. Pooled patient serum (diluted 1:10 (v:v) in blocking buffer) was used as IgE provider to incubate the membrane overnight at 4°C. After using TBST (50 mM Tris‐Cl, 150 mM NaCl, 1% Tween‐20, pH 7.5) to wash the membrane for five times, the membrane was incubated by goat anti‐human IgE‐HRP (catalog A9667, sigma) for 2 hours at 25°C (the goat anti‐human IgE‐HRP was diluted 1:5000 (v:v) in blocking buffer).The results were observed by enhanced chemiluminescense (ECL) detection system (Kangweishiji, Beijing, China).

### 2.6 ELISA essay

Enzyme linked immunosorbent assay, which is known as ELISA, is usually used to quantify the allergenicity of allergens. According to Muraro et al. (2014), peanut proteins were diluted to 10 μg/ml by the carbonate buffer (50 mM, pH 9.6). Then, the samples were added to the microtiter plate by 100 μL per well, after incubated at 4°C for 12 h, the TBS‐Tween buffer were used to wash the wells. Blocking buffer (0.01 mol/L TBS containing 0.1% (w/v) bovine serum, pH 7.5) were utilized to block the plate at 37°C for 1 h and the patient serum (the same serum pool used in Western blots, diluted 1:10 (v:v) in blocking buffer) were added to provide antibodies against Ara h 1. After washed by TBS‐Tween buffer for three times, the horseradish peroxidase‐labeled goat anti‐human IgE (catalog A9667, sigma) was added (diluted 1:10,000 (v:v) in blocking buffer) (100 μL/well) as the secondary antibody. The temperature and time were limited to 37°C and 1 h during the incubation process. 3,3,5,5‐tetramethyl benzidine (TMB) contain 0.03% hydrogen peroxide in 0.1 mol/L PBS was added to each well at 37°C and react for 15 min. H_2_SO_4_ (2 mol/L) were act as the stopper to terminate the reaction. The absorbance was read at 450 nm. For three different samples, the experiments were performed three times and the mean value, as well as the standard deviation, were used for significance analysis.

### 2.7 Protein surface hydrophobic measurement

Fluorescent probe method was used to measure the surface hydrophobic index of protein. According to Li‐Chan & Alizadeh‐Pasdar (2000), protein samples were dissolved in PBS buffer (0.01 mol/L, pH 7.0) and diluted to 0.05, 1.85, 3.65, 5.45, 7.25, 9.05, 10.85 mg/ml. Twenty microliters of 8 mM 8‐benzene amino‐1‐naphthalene sulfonic acid (ANS) were mixed with 2 ml of different protein samples separately. The fluorescence intensity were detected by the fluorescence spectrophotometer at 390 nm. For three different samples, the measurements were performed three times separately. The mean value and standard deviation were used for significance analysis and presented in the bar chart.

### 2.8 Intrinsic fluorescence spectroscopy of Ara h 1

The intrinsic fluorescence spectroscopy of the proteins were detected by a Dual‐FL fluorescence spectrophotometer (HORIBA, Japan) to monitor the changes occurred in the tertiary structure of protein. The concentration of protein sample was 1.00 mg/mL. The excitation wavelength is 280 nm and scanning interval ranged from 250 to 800 nm. The maximum emission wavelength was measured by the supporting software (HORIBA, Japan). For different samples, the measurements were performed three times. The mean value, as well as the standard deviation, were used for significance analysis and presented in the bar chart.

### 2.9 Circular dichroism spectra

A Chirascan spectroscope (Applied Photophysics Ltd, England) was used for CD measurements at room temperature to detect the changes occurred in the secondary structure of protein. According to Vissers et al. (2011), the concentration of protein sample was 1.00 mg/ml. The scanning interval ranged from 190 to 250 nm, the scanning speed is 500 nm/min, and the accumulation frequency is 3. The proportion of typical structures like α‐helixes, β‐sheets, β‐turns, and irregular coils were analyzed by CDNN software (Applied Photophysics Ltd, England). For three different samples, the measurements were performed three times separately. The mean value which corrected with standard deviation was presented in the circular dichroism of Ara h 1 extracted from raw/heat‐treated peanuts.

### 2.10 Statistical analysis

The statistical analysis was performed using SPSS Software (v15.0, SPSS Inc., Chicago, IL, USA). Analysis of variance (ANOVA) was used to determine significant differences between the results and Duncan's test was used to separate the mean with a significance level of 0.05.

## 3 RESULTS

### 3.1 The extraction results of crude and heat‐treated peanuts

**Figure 1:**
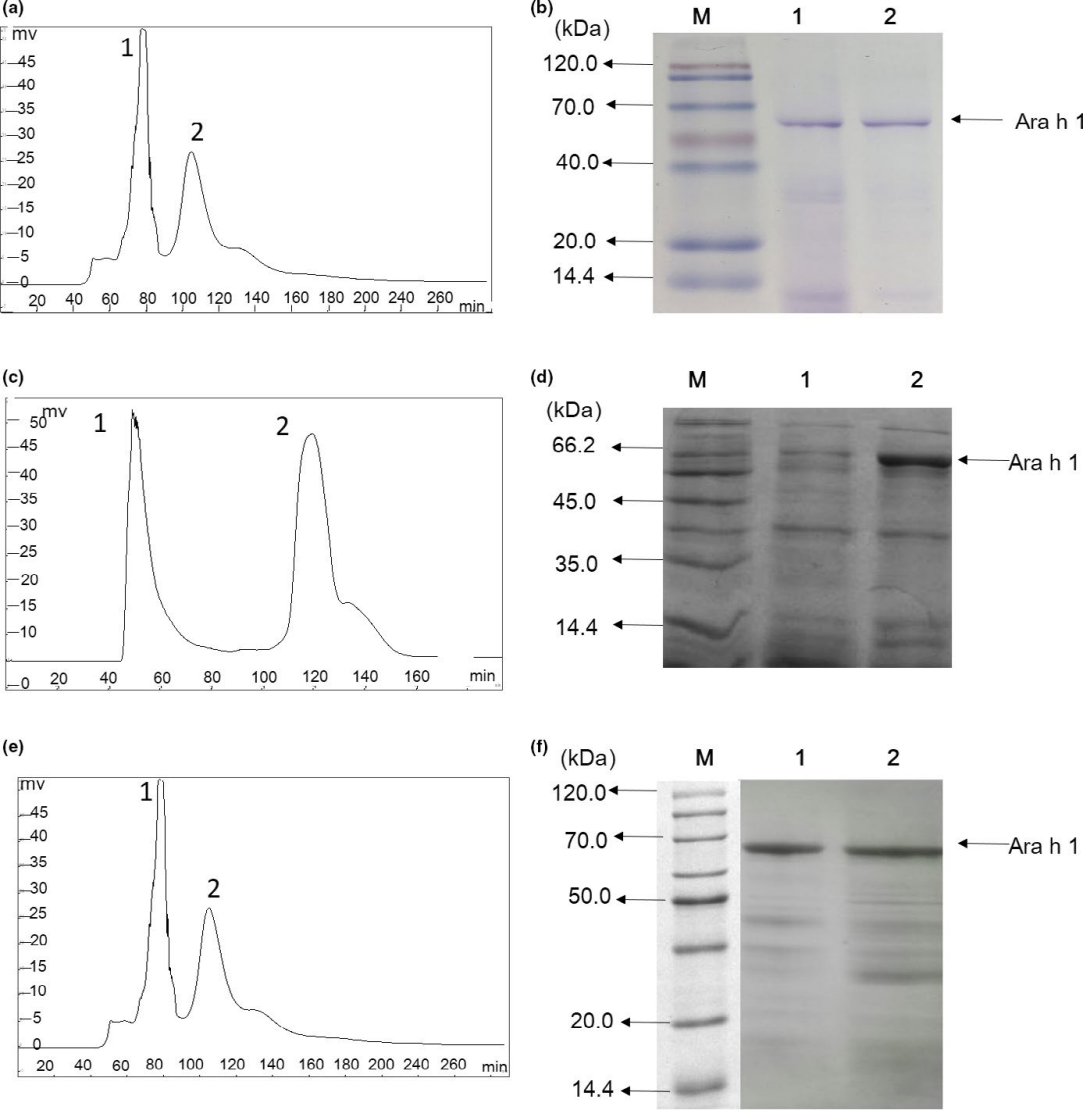
(a) The anion exchange chromatography of Ara h 1 from crude peanuts using DEAE Fast Flow as column (1.6 cm * 25 cm). (b) The SDS‐PAGE analysis of the fractions, the letter M represent the protein maker, the numbers 1–2 represent the fractions collected from chromatography. (c) The anion exchange chromatography of Ara h 1 from boiled peanuts. (d) The SDS‐PAGE analysis of the corresponding fractions. (e) The anion exchange chromatography of Ara h 1 from fried peanuts. (f) The SDS‐PAGE analysis of collected fractions

It can be seen in Figure 1(a), there are two peaks appeared in the anion exchange chromatography spectrum. The first peak started from the 65th minute of elution process, and lasted for 17 min. The second peak started from the 100th minute to the 120th minute. Eluted protein was identified by SDS‐PAGE. From the results in Figure 1(b), obviously there is abundant Ara h 1 appeared in the peak 1 and peak 2. But there are bands (20–40 kDa, <14 kDa) also existed in peak 1, indicating that the protein collected was less pure. According to Figure 1(b) lane 2, Ara h 1 was purified efficiently in peak 2.

In Figure 1(c), there were two peaks appeared in the purification process of Ara h 1 from boiled peanuts. The distance between the two peaks was relatively bigger than which in Figure 1(a). According to Figure 1(d), there was abundant Ara h 1 existed in peak 2, and small fractions with low molecular weight (approximately 35 kDa and 18 kDa), appeared in the same eluted buffer. Similar to Figure 1(a), the two peaks appeared in Figure 1(e) were close to each other, and Ara h 1 were detected in peaks 1 and 2 (Figure 1(f)).

### 3.2 The structural changes in Ara h 1 before and after heat treatment

The hydrophobicity index was measured to characterize the degeneration of protein. The fluorescence spectrum and circular dichroism were used to monitor the partial tertiary and secondary structural changes of protein. And the allergenicity of Ara h 1 was identified by western blot and quantified by ELISA.

#### 3.2.1 The surface hydrophobicity change of Ara h 1

**Figure 2:**
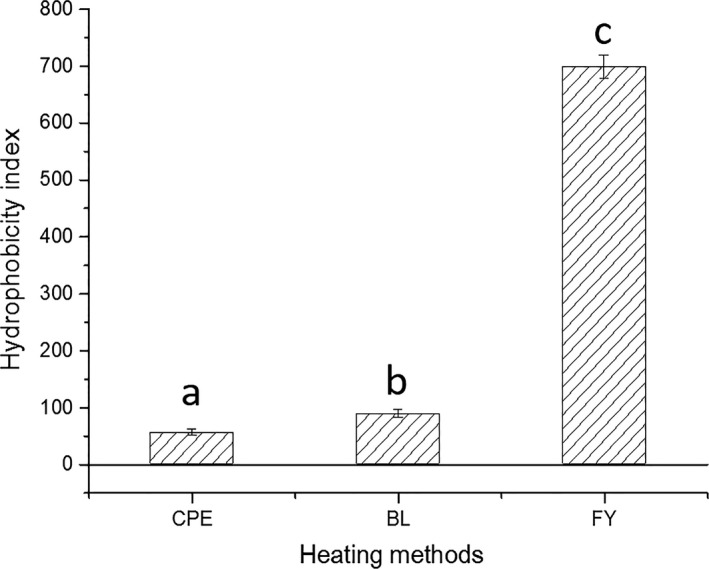
Changes in hydrophobic index of boiled and fried peanuts. CPE represents the crude peanut protein, BL and FY represent Ara h 1 extracted from boiled and fried peanuts, respectively

As it is seen in Figure 2, compared with crude Ara h 1, the hydrophobic index showed slight increase (rising from 52 to 89) in Ara h 1 extracted from boiled peanuts. Besides, approximately a 13‐fold of increase (rising from 52 to 683) appeared in the hydrophobic index of Ara h 1 extracted from fried peanuts, indicating that frying altered the quaternary structure of Ara h 1 significantly.

#### 3.2.2 The changes of partial tertiary structure in Ara h 1

**Figure 3:**
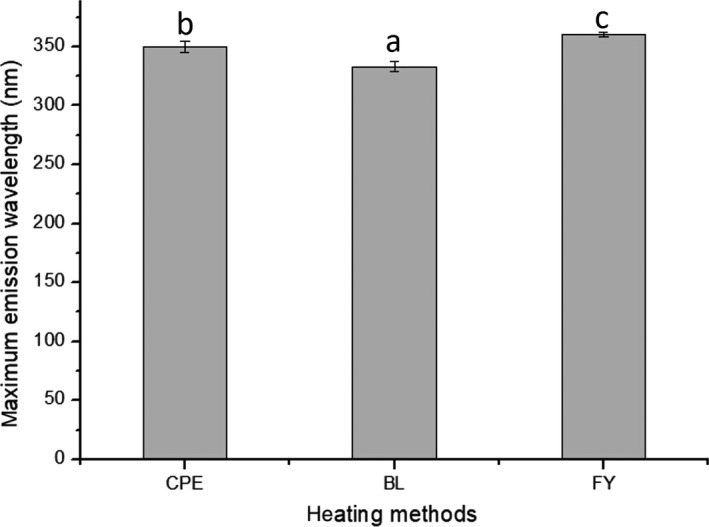
The influence of heat treatment on the fluorescence spectrum of Ara h 1. CPE represents the crude peanut protein, BL and FY represent Ara h 1 extracted from boiled and fried peanuts, respectively

As it is seen in Figure 3, when excited in 280 nm, the signals emitted by Ara h 1 can be detected in 300–400 nm. The alterations in maximum emission wavelength (MEW) can indicate the tertiary structure change of Ara h 1. According to Figure 3, there is a 17‐nm blue shift in the MEW of Ara h 1 extracted from boiled peanut, indicating that the polarity of the microenvironment around the tryptophan is decreased. This may be because of the fractions of Ara h 1 aggregated upon boiling; meanwhile, tryptophan residues gathered in the inner region of the protein. The red shift, together with the leap in hydrophobic index, illustrates that the structure of Ara h 1 was significantly damaged during frying.

#### 3.2.3 The change in secondary structure in Ara h 1 before and after heating

**Figure 4:**
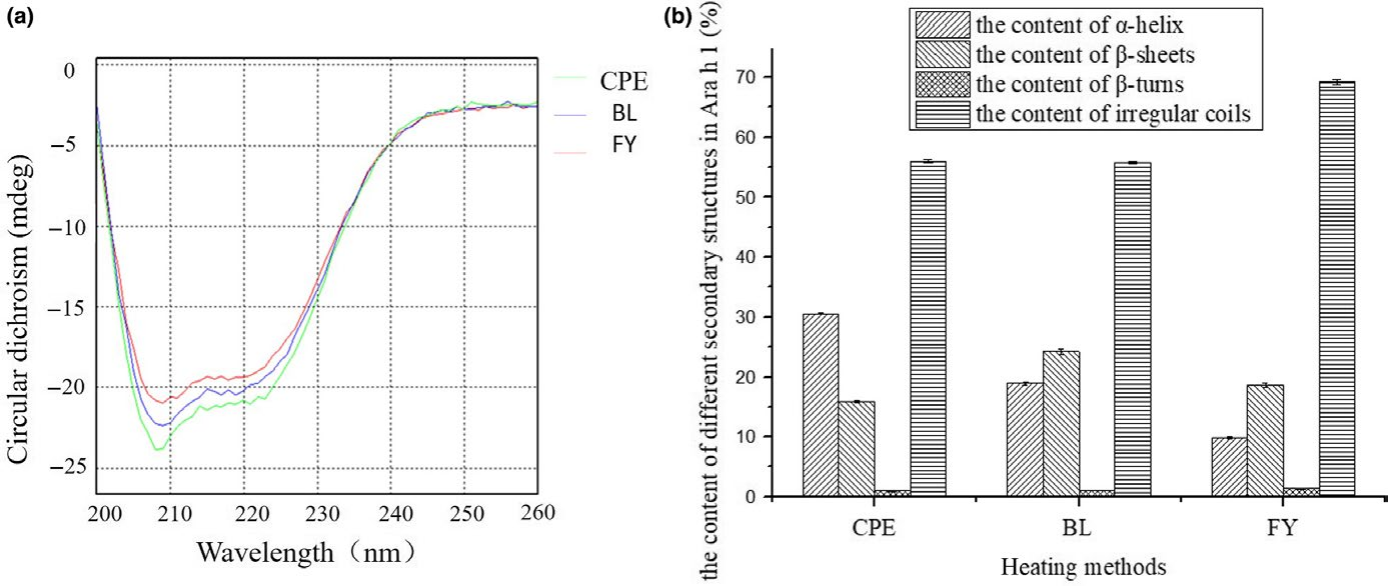
(a) The influence of heat treatment on the circular dichroism of Ara h 1. (b) The effect of heat treatment on the secondary structure of Ara h 1, CPE represents the crude peanut protein, BL and FY represent Ara h 1 extracted from boiled and fried peanuts, respectively

According to Figure 4(a), there are typical α‐helixes in crude and heat‐treated Ara h 1. After the analysis by CDNN software, the proportion of α‐helixes, β‐sheet, β‐turns, and irregular coils are presented in Figure 4(b).

In crude Ara h 1, the content of conserved structure, including α‐helixes and β‐sheets, is accounting for 46.37% among the whole protein. Compared with unheated Ara h 1, the content of α‐helixes in Ara h 1 extracted from boiled peanuts decreased from 30.08% to 19.76%. A 21.06% decline happened in the content of α‐helixes of Ara h 1 extracted from fried peanuts, when compared with raw Ara h 1. The content of β‐sheets increased in Ara h 1 extracted from boiled and fried peanuts, rose to 7.42% and 2.04% separately. It is noteworthy that the content of irregular coils in Ara h 1 extracted from fried peanuts increased to the highest point among the three kinds of Ara h 1s.

### 3.3 The allergenicity changes in Ara h 1 when subjected with heat treatment

**Figure 5:**
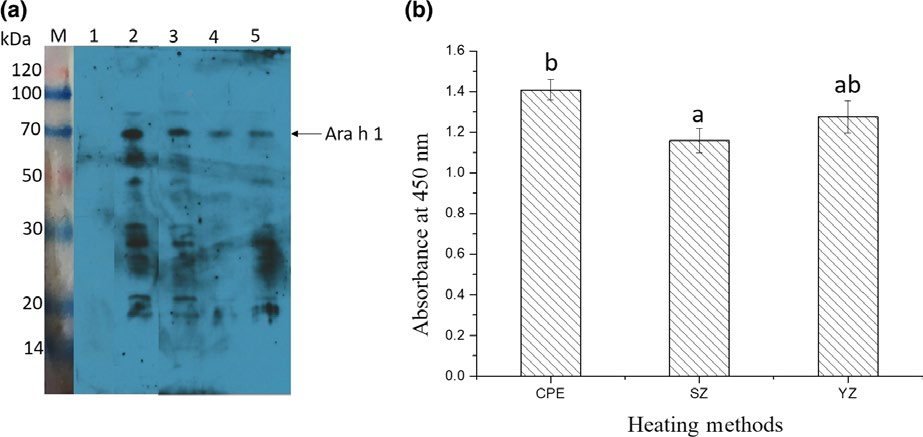
(a) The Western blotting analysis of purified Ara h 1 extracted from fried/crude/boiled peanuts (lines 3–5), letter M represents the protein maker, negative control serum binding with peanut allergens was presented in lane 1, total peanut protein binding with patients' serum was presented in line 2. (b) The ELISA assay of purified Ara h 1, which extracted from raw/boiled/fried peanuts. CPE represents the crude peanut protein, BL and FY represent Ara h 1 extracted from boiled and fried peanuts, respectively

As seen in Figure 5(a), the IgE‐binding capacity of Ara h 1 (extracted from crude, fried, and boiled peanuts) remained after purification, indicating that the DEAE Fast Flow column were suitable for the purification of Ara h 1. According to Figure 5(b), the allergenicity of Ara h 1 decreased when the peanuts were subjected to heat treatment. Compared with crude peanuts, Ara h 1 in boiled peanuts had a much lower allergenicity (Figure 5b). The IgE‐binding capacity of Ara h 1 in fried peanuts decreased 9.40% compared to the crude Ara h 1 (Figure 5b).

## 4 DISCUSSION

As well as oxidation, thermal processing can induce the rise of surface hydrophobic index of proteins, because of the exposure of the inner hydrophobic residues (Sriket, Benjakul, Visessanguan, & Kijroongrojana, 2007). According to Figure 2, the hydrophobic index rises remarkably in fried peanuts, indicating the release of polar amino acids during frying process, as well as the formation of cross‐linking between the aromatic amino acids and allergens. It has been reported that the solubility of Ara h 1 decreased in fried peanuts, due to the aggregation effect of subunits and monomers of Ara h 1 (Beyer et al., 2001).

Approximately 90% of the allergenicity of hazelnuts will be eliminated after treated with heat (Skamstrup Hansen et al., 2003). According to the structural alteration results presented earlier, frying can make the structure of Ara h 1 looser compared to boiling. When oil utilized as medium in the thermal process, the temperatures would be increased rapidly. Conformational epitopes which mapped on the three‐dimensional structure of Ara h 1 can be destroyed during the degradation upon frying process.

Data presented in Figure 4(b) showed that the less α‐helixes (a relatively tight structure in secondary structure) contained in fried Ara h 1, which can indicate the degradation occurred in the Ara h 1 in fired peanuts, is much serious than the one extracted from boiled peanuts. However, instead of maintaining the lowest allergenicity, it is noticeable that the immunoreactivity of Ara h 1 in fried peanuts is higher than which of the boiled peanuts and lower than the crude Ara h 1 (Figure 5(b)). This may because of the strong surface hydrophobic interaction maintained by Ara h 1 in fried peanuts (Figure 2(a)) enhanced the binding capacity between the allergen and IgE. Furthermore, the slightly increased content of β‐sheets (increased from 15.82% to 18.63%) appeared in Ara h 1 from fried peanuts (Figure 4(b)) can protect the epitopes from losing and destroying (Blanc et al., 2011).

Ara h 1 in boiled peanuts was assumed to be structurally changed in a mild way (compared with frying). The allergenicity is the lowest among the three Ara h 1s, this may be because the epitopes were transformed into the cavity of the protein due to the hydrophobic interaction. After attacked by water molecules, the small fractions of Ara h 1 would transfer into the cooking water and thus epitopes were destroyed during boiling process. Furthermore, aggregates formed upon boiling cannot be dissolved in water and therefore can hinder the Maillard reaction from happening and consequently avoid the formation of new epitopes (Blanc et al., 2011; Schmitt, Nesbit, Hurlburt, Cheng, & Maleki, 2010). In general, Ara h 1 in boiled peanuts has the lowest sensitivity due to the decreased protein content and structural changes occurred in boiling.

The increase of hydrophobicity index of Ara h 1 extracted from boiled peanuts (Figure 2) was probably due to the degradation effect of protein fractions. It has been reported that hydrophobic groups were released into the reaction system during the initial process of boiling. And then the degraded polypeptides, which produced in the earlier stage of heating process, were assembled into much larger aggregated structures when the heating time reaches 20 min (Tian, Rao, Tao, & Xue, 2018). Polypeptides containing tryptophan were estimated to be transformed into the cavity of Ara h 1 upon the aggregation and thus caused a blue shift in the fluorescence emission spectra. In Figure 1(c), it is obvious that the distance between the two peaks was larger than which in the Figure 1(a) and (e), it can be assumed that the aggregates of Ara h 1 (extracted from boiled peanuts) had increased electric charge when compared with native Ara h 1. The peak area of peak 2 appeared in Figure 1(c) was bigger than corresponding peaks appeared in Figure 1(a) and (e), assuming that the content of aggregates formed during boiling were at a higher level than crude and fried peanuts.

It can be deducted that the change of allergenicity is unpredictable due to the variable thermal process and its conditions. On the one hand, thermal process can lower the amount of allergens. For example, the content of Ara h 1 decreased approximately 11.5 folds of the control after treated with heat (Dileepan et al., 2014). On the other hand, the chemical changes and modifications like decomposition, aggregation, and rearrangement can form new epitopes appeared on the surface of protein and thus increase the sensitivity of allergens (Beyer et al., 2001; Guillon, Bernard, Drumare, Hazebrouck, & Adel‐Patient, 2016; Moghaddam et al., 2014).

Besides, many allergens are reported to be resistant to digestion, this characteristic has been listed as a criterion to distinguish between potentially allergenic and nonallergenic proteins (Koppelman, Hefle, Taylor, & De Jong, 2010). Allergens digested by pepsin or trypsin and then transformed into low molecular peptides, which maintain antigenic determinants, are still have the potential to cause sensitive reactions in the human body. Although it has been shown Ara h 1 extracted from fried peanuts has a relatively looser structure than the other two Ara h 1s (Figures 2, 4(b)), the allergenicity did not decreased significantly when compared with Ara h 1 extracted from crude peanuts. This maybe because of the linear epitopes of Ara h 1 (extracted from fried peanuts) were not destroyed during the frying and maintained a high IgE‐binding capacity. Conformational epitopes disrupted during thermal processing can explain the slightly decreased allergenicity of Ara h 1 extracted from boiled and fried peanuts.

In this study, instead of imposing thermal processing to the purified Ara h 1, we extract Ara h 1 from boiled and fried peanuts, aiming to mimic the thermal process as well as monitoring the specific changes occurred to Ara h 1. Water, oil, and other medium participate in the thermal process effectively and sometimes react with allergens contained in peanuts (Alves et al., 2017). In the previous studies, researchers isolated Ara h 1, Ara h 2, Ara h 6, or other allergens and which were then modified by glucose to imitate the Millard reaction occurred (Mueller et al., 2013; Vissers et al., 2011). The diversity and complexity of thermal process are neglected to a certain degree, and it is necessary to explore the structural and sensitivity change of allergens extracted from heat‐treated peanuts.

## 5 CONCLUSION

To clarify the connections between heat treatment and allergenicity of Ara h 1, we analyzed the structural changes of the allergen. Ara h 1 was isolated from crude, boiled, and fried peanuts successively by anion exchange chromatography. Compared with boiling, frying can alter the three‐dimensional structure of Ara h 1. Ara h 1 in boiled peanuts presented degradation and aggregation during different period of boiling. Besides, boiling could help the allergenicity of Ara h 1 to reach the bottom point among the three kinds of Ara h 1 we tested.

## CONFLICT OF INTEREST

The authors declare that they do not have any conflict of interest.

## ETHICAL REVIEW

This study was approved by the Review Board of China Agricultural University.

## INFORMED CONSENT

Written informed consent was obtained from all study participants.
